# Wavelengths and Energy Levels of Neutral Kr^84^ and Level Shifts in All Kr Even Isotopes

**DOI:** 10.6028/jres.098.047

**Published:** 1993

**Authors:** Victor Kaufman

**Affiliations:** National Institute of Standards and Technology, Gaithersburg, MD 20899-0001

**Keywords:** energy levels, interferometry, isotope shift, krypton, wavelengths

## Abstract

Interferometrically-measured wavelengths of 109 lines of neutral Kr^84^ are compared with those of Kr^86^. Sixty energy levels of neutral Kr^84^ derived from those wavelengths and 25 Kr^86^–Kr^84^ isotope shifts previously measured are given along with their shifts from the energy levels of Kr^86^. Twenty levels of each of Kr^82^, Kr^80^, and Kr^78^ are also evaluated using isotope-shift information in the literature. The differences between the experimentally observed shifts and the normal mass shift leave large negative residuals which are accounted for by ionization energy differences and by the specific mass shift. It appears that the volume effect causes only a very small, if any, energy level shift.

## 1. Introduction

In 1969, Kaufman and Humphreys [1] determined a set of 45 even-parity and 66 odd-parity levels of neutral Kr^86^ based upon interferometrically-determined wavelengths of that isotope of krypton. The energies relative to the ground level, based on the vacuum-ultraviolet observations of Petersson [2], had an absolute uncertainty of ±0.15 cm^−1^, although their relative uncertainties were much smaller. (All uncertainties in this paper are one standard deviation estimates unless otherwise stated.) Trickl et al. [3], in 1989, measured the values of the resonance lines of Kr^86^ from four of the *J* = 1 levels of the 4*p*^5^5*s*, 6*s*, and *7s* configurations with an average uncertainty of 1 part in 10^7^. Their results led to a subtraction of (0.0679±0.0061) cm^−1^ from the values given by Kaufman and Humphreys [1]. This correction was incorporated in the compilation of the energy levels by Sugar and Musgrove [4].

## 2. Wavelengths of Neutral Kr^84^

At the same time that the author made interferometric wavelength measurements of the Kr^86^ transitions, he also did the same for a number of lines of Kr^84^ I between 4264 Å and 6906 Å. The pure isotope lamps were of the hot cathode type devised by Kösters and Engelhard at the Physikalisch-Technische Bundesanstalt (PTB). They were operated in the manner recommended by the International Commission of Weights and Measures [5].

During the summer of 1967, the author was given the opportunity to spend some time with C. J. Humphreys at the Naval Weapons Center Corona Laboratories, Corona, CA. With the cooperation of Dr. Humphreys and his staff, measurements were made on Kr^84^ wavelengths between 7589 Å and 9754 Å. The orange line of Kr^86^ at 6057 Å, as emitted from a microwave-excited electrodeless discharge lamp maintained in a bath of nitrogen at its triple-point temperature, was used as the wavelength standard. The Kr^84^ spectrum was observed from a similarly cooled, microwave-excited electrodeless lamp. Both lamps were viewed along the capillary. Interferometer spacers of 50 mm, 80 mm, and 100 mm were used, but final corrections for dispersion of phase change were taken from the more extensive data of C. J. Humphreys.

The 109 interferometricaliy measured vacuum wavelengths of Kr^84^ are given in the first column of Table 1. Also included in the table are the wavelength uncertainties in parentheses (in units of the last digit) and wavenumbers of the Kr^84^ lines, the Kr^86^ wavelengths and wavenumbers for those same 109 lines from Ref. [1], the classification of each transition and the wavenumber difference between the two isotopes. The wavelength uncertainties are the rms deviations in the set of measurements for each line.

A number of investigators [6–13] have made direct measurements of isotope shifts between Kr^86^ and Kr^84^. These are also given in Table 1. Direct measurements, by their very nature, are much more accurate than the difference between two completely independent measurements. This is evident in the stated uncertainties following both the differences, Δ*ν*, and the direct isotope separations in Table 1. However, it should be noted that there is agreement within the stated uncertainties in most cases.

## 3. Energy Levels of Kr^84^

Trickl et al. [3] also measured the isotope shifts in the aforementioned four vacuum-ultraviolet lines for several of the isotopes of krypton, including those between Kr^86^ and Kr^84^. Without those measurements, it would not be possible to give separate values for the energy levels of the two isotopes relative to the ground level.

With the aid of the same iterative level-calculation program used in Ref. [1], the 109 wavenumbers of Kr^84^ given in Table 1 were combined with the Kr^86^–Kr^84^ isotope shift measurements from Refs. [6–13] and with the vuv values for Kr^84^ from Ref. [3] to determine the Kr^84^ energy-level values. Table 2 includes these newly determined values with their uncertainties (in units of the last digit) in parentheses. Also included are the Kr^86^ values for these same levels from the work of Kaufman and Humphreys [1], the Kr^86^–Kr^84^ differences and the number of transitions to or from each Kr^84^ level included in the 109 wavelength measurements.

Jackson [6,13] measured wavenumber shifts between pairs of the five stable even isotopes of krypton in fifteen 5*s–*5*p* lines, six 5*s–*6*p* lines, and one 5*p–*6*d* line. Champeau and Keller [8] did the same for two other 5*s–*5*p* transitions and one 5*s–*6*p* transition for all but the Kr^78^ isotope. Further information on four of these 25 lines can be found in Refs. [7] and [9–12], Combining these previously reported data with the vuv high resolution measurements of the isotope shifts between Kr^86^ and each of Kr^82^, Kr^80^, and Kr^78^ given by Trickl et al. [3] for the 4*p*^6^*–*4*p*^5^ (^2^P°_1/2_)5*s* ^2^[l/2]°_1_ transition, it is possible to evaluate a total of 20 excited levels in each of these isotopes. These values are given in Table 3. The isotope shifts of these levels with respect to those of Kr^86^ are also given.

Discussions of the two effects, the finite mass of the nucleus and the non-zero nuclear volume, which lead to the observation of isotope shifts are discussed in some detail in Refs. [3, 6 and 13]. In all of those instances, the authors point out that the difference between the experimentally observed isotope shift of a *line*, Δ*ν*_diff_, and the normal mass shift, Δ*ν*_nm_, is accounted for by the sum of the specific mass shift, Δ*ν*_sm_, and the shift due to the volume effect, Δ*ν*_vol_. We now have the opportunity to discuss the difference in the term values of the *levels* of isotopes. The term value *T* is defined as the binding energy of the electron, i.e., *T = IE* − (Level value). Here *IE* is the energy of the appropriate Kr II 4*p*^5 2^P°_3/2_ or ^2^P_1/2_ level with respect to the Kr I 4*p*^6 1^S_0_ ground level, which has been taken as zero for each isotope. The normal mass shifts of the term values of levels near the ionization limit approach zero and increase with increasing term value of a level. The values of Δ*ν*_nm_ for the corresponding term-value differences are given for Kr^86^–Kr^84^ in column 4 of Table 2 and for Kr^86^–Kr^82^, Kr^86^–Kr^80^, and Kr^86^–Kr^78^ in Table 3. They show that the differences in binding energies of the 4*p*^6 1^S_0_ ground states due only to the normal mass effect are 17.0× 10^−3^, 34.9×10^−3^, 53.6×10^−6^, and 73.3×10^−3^ cm^−1^, respectively for Kr^84^, Kr^82^, Kr^80^, and Kr^78^ with respect to Kr^86^.

For any two isotopes

$$\Delta T_{\exp}=\Delta \left(IE\right)-\Delta \nu_{\text{lev}}=\Delta \nu_{\text{nm}}+\Delta \nu_{\text{sm}}+\Delta \nu_{\text{vol}}$$ (1)

where Δ*T*_exp_ is the observed shift in the term value. Thus by adding the experimentally observed isotope shift Δ*ν*_lev_ and the calculated normal mass shift Δ*ν*_nm_ for term values of levels relatively close to the ionization limit, where the specific mass and volume effects must be negligible, we can find values of the differences in the 4*p*^6^−4*p*^5 2^P^o^ ionization energies of these isotopes. The addition of columns 3 and 4 of Table 2 for Kr^86^–Kr^84^ and the equivalent columns in Table 3 for the other three krypton pairs, gives ionization energy differences with estimated uncertainties of ±0.5×10^−3^ cm^−1^ of these four isotopes from Kr^86^. They are 7.5×10^−3^, 15.6×10^−3^, 24.1×10^−3^, and 32.4×10^−3^ cm^−1^, respectively for Kr^84^, K^82^ Kr^80^, and Kr^78^. By definition, these are the experimental differences between the binding energies of a 4*p* electron in the ground level of these respective isotopes and the same binding energy for the Kr^86^ isotope.

Table 4 gives the term value shifts due to the sum of the specific mass and volume effects as obtained by applying Eq. (1) to the 4*p*^6 1^S_0_ and the other twenty levels given in Table 3 and the same levels in Table 2. The estimated uncertainty of ±0.6×10^−3^ cm^−1^ for term value shifts is due to both the estimated uncertainty of ±0.5×10^−3^ cm^−1^ on the ionization energy differences given above and an uncertainty of about 0.2×10^−3^ cm^−1^ on the value of the isotope shifts.

## Figures and Tables

**Table 1:** Interferometricaliy measured vacuum wavelengths of Kr^84^I and differences from those of Kr^86^I

Wavelength (Å)		Wavenumber (cm^−1^)		Classification					Δ*ν*_diff_^b^	Previous measurements^c^		
Kr^84^	Kr^86^	Kr^84^	Kr^86^	odd level			Even level					
4264.4856(3)^a^	.48475	23449.4871	.4918	$\left({\;}^{2}P_{1/2}^{o}\right)5s$	${\;}^{2}{\left[1/2\right]}_{1}^{o}$	–	$\left({\;}^{2}P_{3/2}^{o}\right)8p$	^2^[1/2]_0_	4.7			
4275.17220(10)	.17148	23390.8707	.8746	$\left({\;}^{2}P_{3/2}^{o}\right)5s$	${\;}^{2}{\left[3/2\right]}_{2}^{o}$	–	$\left({\;}^{2}P_{3/2}^{o}\right)6p$	^2^[3/2]_2_	3.9(5)	3.93(8)^d^		
4284.17248(10)	.17172	23341.7306	.7348	$\left({\;}^{2}P_{3/2}^{o}\right)5s$	${\;}^{2}{\left[3/2\right]}_{2}^{o}$	–	$\left({\;}^{2}P_{3/2}^{o}\right)6p$	^2^[3/2]_1_	4.2			
4287.69293(10)	.69212	23322.5657	.5701	$\left({\;}^{2}P_{1/2}^{o}\right)5s$	${\;}^{2}{\left[1/2\right]}_{0}^{o}$	–	$\left({\;}^{2}P_{1/2}^{o}\right)6p$	^2^[1/2]_1_	4.4			
4301.69626(10)	.69545	23246.6436	.6480	$\left({\;}^{2}P_{1/2}^{o}\right)5s$	${\;}^{2}{\left[1/2\right]}_{0}^{o}$	–	$\left({\;}^{2}P_{1/2}^{o}\right)6p$	^2^[3/2]_1_	4.4			
4319.7658(3)	.76561	23149.4032	.4042	$\left({\;}^{2}P_{3/2}^{o}\right)5s$	${\;}^{2}{\left[3/2\right]}_{2}^{o}$	–	$\left({\;}^{2}P_{3/2}^{o}\right)6p$	^2^[5/2]_2_	1.0			
4320.7942(3)	.79361	23143.8933	.8965	$\left({\;}^{2}P_{3/2}^{o}\right)5s$	${\;}^{2}{\left[3/2\right]}_{2}^{o}$	–	$\left({\;}^{2}P_{3/2}^{o}\right)6p$	^2^[5/2]_3_	3.2(1.6)	3.90(8)^d^	3.86(3)^e^	
4352.58282(10)	.58189	22974.8644	.8693	$\left({\;}^{2}P_{1/2}^{o}\right)5s$	${\;}^{2}{\left[1/2\right]}_{1}^{o}$	–	$\left({\;}^{2}P_{1/2}^{o}\right)6p$	^2^[1/2]_0_	4.9			
4363.86767(10)	.86695	22915.4520	.4558	$\left({\;}^{2}P_{3/2}^{o}\right)5s$	${\;}^{2}{\left[3/2\right]}_{2}^{o}$	–	$\left({\;}^{2}P_{3/2}^{o}\right)6p$	^2^[1/2]_1_	3.8(5)	3.84(7)^d^		
4377.35124(10)	.35027	22844.8654	.8705	$\left({\;}^{2}P_{3/2}^{o}\right)5s$	${\;}^{2}{\left[3/2\right]}_{1}^{o}$	–	$\left({\;}^{2}P_{3/2}^{o}\right)6p$	^2^[1/2]_0_	5.1(5)	4.90(8)^d^		
4401.20227(10)	.20109	22721.0643	.0704	$\left({\;}^{2}P_{1/2}^{o}\right)5s$	${\;}^{2}{\left[1/2\right]}_{1}^{o}$	–	$\left({\;}^{2}P_{1/2}^{o}\right)6p$	^2^[3/2]_2_	6.1			
4411.6068(2)	.60581	22667.4780	.4831	$\left({\;}^{2}P_{1/2}^{o}\right)5s$	${\;}^{2}{\left[1/2\right]}_{1}^{o}$	–	$\left({\;}^{2}P_{1/2}^{o}\right)6p$	^2^[1/2]_1_	5.1			
4420.0022(3)	.00126	22624.4231	.4279	$\left({\;}^{2}P_{1/2}^{o}\right)5s$	${\;}^{2}{\left[1/2\right]}_{1}^{o}$	–	$\left({\;}^{2}P_{3/2}^{o}\right)5f$	^2^[3/2]_2_	4.8			
4426.43265(10)	.43162	22591.5558	.5610	$\left({\;}^{2}P_{1/2}^{o}\right)5s$	${\;}^{2}{\left[1/2\right]}_{1}^{o}$	–	$\left({\;}^{2}P_{1/2}^{o}\right)6p$	^2^[3/2]_1_	5.2			
4455.16765(10)	.16670	22445.8444	.8492	$\left({\;}^{2}P_{3/2}^{o}\right)5s$	${\;}^{2}{\left[3/2\right]}_{1}^{o}$	–	$\left({\;}^{2}P_{3/2}^{o}\right)6p$	^2^[3/2]_2_	4.8			
4464.94273(10)	.94163	22396.7038	.7094	$\left({\;}^{2}P_{3/2}^{o}\right)5s$	${\;}^{2}{\left[3/2\right]}_{1}^{o}$	–	$\left({\;}^{2}P_{3/2}^{o}\right)6p$	^2^[3/2]_1_	5.6(5)	4.83(7)^d^		
4503.61721(10)	.61621	22204.3738	.3787	$\left({\;}^{2}P_{3/2}^{o}\right)5s$	${\;}^{2}{\left[3/2\right]}_{1}^{o}$	–	$\left({\;}^{2}P_{3/2}^{o}\right)6p$	^2^[5/2]_2_	4.9(5)	4.76(8)^d^		
5217.2701(3)	.2695	19167.1119	.1141	$\left({\;}^{2}P_{3/2}^{o}\right)5p$	^2^[1/2]_1_	–	$\left({\;}^{2}P_{3/2}^{o}\right)8d$	${\;}^{2}{\left[1/2\right]}_{0}^{o}$	2.2			
5229.63279(10)	.63192	19121.8015	.8047	$\left({\;}^{2}P_{3/2}^{o}\right)5p$	^2^[1/2]_1_	–	$\left({\;}^{2}P_{3/2}^{o}\right)8d$	${\;}^{2}{\left[1/2\right]}_{1}^{o}$	3.2			
5281.3038(2)	.30263	18934.7186	.7226	$\left({\;}^{2}P_{3/2}^{o}\right)5p$	^2^[1/2]_1_	–	$\left({\;}^{2}P_{1/2}^{o}\right)5d$	${\;}^{2}{\left[3/2\right]}_{2}^{o}$	4.0			
5336.2389(3)	.23880	18739.7907	.7910	$\left({\;}^{2}P_{3/2}^{o}\right)5p$	^2^[5/2]_2_	–	$\left({\;}^{2}P_{3/2}^{o}\right)9d$	${\;}^{2}{\left[7/2\right]}_{3}^{o}$	0.3			
5340.6034(2)	.60296	18724.4760	.4775	$\left({\;}^{2}P_{3/2}^{o}\right)5p$	^2^[5/2]_3_	–	$\left({\;}^{2}P_{3/2}^{o}\right)9d$	${\;}^{2}{\left[7/2\right]}_{4}^{o}$	1.5			
5381.1325(2)	.13155	18583.4487	.4520	$\left({\;}^{2}P_{3/2}^{o}\right)5p$	^2^[1/2]_1_	–	$\left({\;}^{2}P_{3/2}^{o}\right)9s$	${\;}^{2}{\left[3/2\right]}_{2}^{o}$	3.3			
5492.46155(10)	.46062	18206.7729	.7760	$\left({\;}^{2}P_{3/2}^{o}\right)5p$	^2^[1/2]_1_	–	$\left({\;}^{2}P_{3/2}^{o}\right)7d$	${\;}^{2}{\left[3/2\right]}_{2}^{o}$	3.1			
5502.23831(10)	.23721	18174.4218	.4255	$\left({\;}^{2}P_{3/2}^{o}\right)5p$	^2^[1/2]_1_	–	$\left({\;}^{2}P_{3/2}^{o}\right)7d$	${\;}^{2}{\left[1/2\right]}_{1}^{o}$	3.7			
5505.5344(3)	.5339	18163.5410	.5427	$\left({\;}^{2}P_{3/2}^{o}\right)5p$	^2^[5/2]_2_	–	$\left({\;}^{2}P_{3/2}^{o}\right)8d$	${\;}^{2}{\left[7/2\right]}_{3}^{o}$	1.7			
5505.8596(3)	.8591	18162.4682	.4699	$\left({\;}^{2}P_{3/2}^{o}\right)5p$	^2^[1/2]_1_	–	$\left({\;}^{2}P_{3/2}^{o}\right)7d$	${\;}^{2}{\left[1/2\right]}_{0}^{o}$	1.7			
5518.1990(4)	.19756	18121.8546	.8593	$\left({\;}^{2}P_{1/2}^{o}\right)5s$	${\;}^{2}{\left[1/2\right]}_{0}^{o}$	–	$\left({\;}^{2}P_{3/2}^{o}\right)6p$	^2^[3/2]_1_	4.7			
5522.04373(10)	.04304	18109.2373	.2395	$\left({\;}^{2}P_{3/2}^{o}\right)5p$	^2^[5/2]_3_	–	$\left({\;}^{2}P_{3/2}^{o}\right)8d$	${\;}^{2}{\left[7/2\right]}_{4}^{o}$	2.2			
5563.76997(10)	.76905	17973.4246	.4276	$\left({\;}^{2}P_{3/2}^{o}\right)5s$	${\;}^{2}{\left[3/2\right]}_{2}^{o}$	–	$\left({\;}^{2}P_{1/2}^{o}\right)5p$	^2^[3/2]_2_	3.0(3)	2.88(5)^f^	2.86(3)^g^	
5571.83623(10)	.83525	17947.4048	.4079	$\left({\;}^{2}P_{3/2}^{o}\right)5s$	${\;}^{2}{\left[3/2\right]}_{2}^{o}$	–	$\left({\;}^{2}P_{1/2}^{o}\right)5p$	^2^[1/2]_1_	3.1(3)	2.85(10)^f^	2.87(3)^g^	2.85(2)^h^,^i^
5581.93678(10)	.93533	17914.9288	.9335	$\left({\;}^{2}P_{1/2}^{o}\right)5s$	${\;}^{2}{\left[1/2\right]}_{1}^{o}$	–	$\left({\;}^{2}P_{3/2}^{o}\right)6p$	^2^[1/2]_0_	4.7			
5613.3678(3)	.3669	17814.6175	.6203	$\left({\;}^{2}P_{3/2}^{o}\right)5p$	^2^[5/2]_2_	–	$\left({\;}^{2}P_{1/2}^{o}\right)5d$	${\;}^{2}{\left[5/2\right]}_{2}^{o}$	2.8			
5651.12975(10)	.12861	17695.5767	.5803	$\left({\;}^{2}P_{1/2}^{o}\right)5s$	${\;}^{2}{\left[1/2\right]}_{0}^{o}$	–	$\left({\;}^{2}P_{3/2}^{o}\right)6p$	^2^[1/2]_1_	3.6(3)	3.33(20)^f^		
5674.0250(2)	.02398	17624.1733	.1765	$\left({\;}^{2}P_{3/2}^{o}\right)5s$	${\;}^{2}{\left[3/2\right]}_{2}^{o}$	–	$\left({\;}^{2}P_{1/2}^{o}\right)5p$	^2^[3/2]_1_	3.2			
5703.7524(3)	.7513	17532.3179	.3212	$\left({\;}^{2}P_{3/2}^{o}\right)5p$	^2^[3/2]_1_	–	$\left({\;}^{2}P_{3/2}^{o}\right)8d$	${\;}^{2}{\left[5/2\right]}_{2}^{o}$	3.3			
5709.0964(2)	.09462	17515.9067	.9122	$\left({\;}^{2}P_{1/2}^{o}\right)5s$	${\;}^{2}{\left[1/2\right]}_{1}^{o}$	–	$\left({\;}^{2}P_{3/2}^{o}\right)6p$	^2^[3/2]_2_	5.5			
5723.4644(4)	.46398	17471.9354	.9366	$\left({\;}^{2}P_{3/2}^{o}\right)5p$	^2^[5/2]_2_	–	$\left({\;}^{2}P_{3/2}^{o}\right)9s$	${\;}^{2}{\left[3/2\right]}_{1}^{o}$	1.2			
5728.1764(4)	.17554	17457.5629	.5656	$\left({\;}^{2}P_{3/2}^{o}\right)5p$	^2^[5/2]_3_	–	$\left({\;}^{2}P_{3/2}^{o}\right)9s$	${\;}^{2}{\left[3/2\right]}_{2}^{o}$	2.7			
5764.4937(4)	.4927	17347.5773	.5803	$\left({\;}^{2}P_{3/2}^{o}\right)5p$	^2^[3/2]_2_	–	$\left({\;}^{2}P_{3/2}^{o}\right)8d$	${\;}^{2}{\left[7/2\right]}_{3}^{o}$	3.0			
5785.4977(4)	.49685	17284J977	.6002	$\left({\;}^{2}P_{3/2}^{o}\right)5p$	^2^[5/2]_3_	–	$\left({\;}^{2}P_{3/2}^{o}\right)7d$	${\;}^{2}{\left[5/2\right]}_{3}^{o}$	2.5			
5807.15097(10)	.14992	17220.1481	.1513	$\left({\;}^{2}P_{3/2}^{o}\right)5p$	^2^[5/2]_2_	–	$\left({\;}^{2}P_{3/2}^{o}\right)7d$	${\;}^{2}{\left[5/2\right]}_{2}^{o}$	3.2			
5821.7315(2)	.73068	17177.0203	.0227	$\left({\;}^{2}P_{3/2}^{o}\right)5p$	^2^[5/2]_3_	–	$\left({\;}^{2}P_{3/2}^{o}\right)7d$	${\;}^{2}{\left[7/2\right]}_{3}^{o}$	2.4			
5826.1333(2)	.13246	17164.0426	.0451	$\left({\;}^{2}P_{3/2}^{o}\right)5p$	^2^[5/2]_2_	–	$\left({\;}^{2}P_{3/2}^{o}\right)7d$	${\;}^{2}{\left[7/2\right]}_{3}^{o}$	2.5			
5834.47363(10)	.47251	17139.5067	.5100	$\left({\;}^{2}P_{3/2}^{o}\right)5p$	^2^[5/2]_3_	–	$\left({\;}^{2}P_{3/2}^{o}\right)7d$	${\;}^{2}{\left[7/2\right]}_{4}^{o}$	3.3			
5854.4974(3)	.49603	17080.8855	.8895	$\left({\;}^{2}P_{3/2}^{o}\right)5p$	^2^[5/2]_3_	–	$\left({\;}^{2}P_{3/2}^{o}\right)7d$	${\;}^{2}{\left[3/2\right]}_{2}^{o}$	4.0			
5868.37627(10)	.37472	17040.4888	.4933	$\left({\;}^{2}P_{3/2}^{o}\right)5s$	${\;}^{2}{\left[1/2\right]}_{1}^{o}$	–	$\left({\;}^{2}P_{3/2}^{o}\right)6p$	^2^[1/2]_1_	4.5			
5872.54320(10)	.54160	17028.3975	.4022	$\left({\;}^{2}P_{3/2}^{o}\right)5s$	${\;}^{2}{\left[3/2\right]}_{1}^{o}$	–	$\left({\;}^{2}P_{1/2}^{o}\right)5p$	^2^[3/2]_2_	4.7(3)	3.77(5)^d^	3.94(20)^j^	
5881.52997(10)	.52867	17002.3787	.3825	$\left({\;}^{2}P_{3/2}^{o}\right)5s$	${\;}^{2}{\left[3/2\right]}_{1}^{o}$	–	$\left({\;}^{2}P_{1/2}^{o}\right)5p$	^2^[1/2]_1_	3.8			
5889.3185(2)	.31753	16979.8933	.8961	$\left({\;}^{2}P_{3/2}^{o}\right)5p$	^2^[3/2]_2_	–	$\left({\;}^{2}P_{1/2}^{o}\right)5d$	${\;}^{2}{\left[3/2\right]}_{2}^{o}$	2.8			
5947.09957(10)	.09902	16814.9194	.9210	$\left({\;}^{2}P_{3/2}^{o}\right)5p$	^2^[3/2]_1_	–	$\left({\;}^{2}P_{3/2}^{o}\right)9s$	${\;}^{2}{\left[3/2\right]}_{1}^{o}$	1.6			
5979.30154(10)	.30061	16724.3614	.3640	$\left({\;}^{2}P_{3/2}^{o}\right)5p$	^2^[3/2]_2_	–	$\left({\;}^{2}P_{3/2}^{o}\right)7d$	${\;}^{2}{\left[3/2\right]}_{1}^{o}$	2.6			
5995.51035(10)	.50899	16679.1473	.1510	$\left({\;}^{2}P_{3/2}^{o}\right)5s$	${\;}^{2}{\left[3/2\right]}_{1}^{o}$	–	$\left({\;}^{2}P_{1/2}^{o}\right)5p$	^2^[3/2]_1_	3.7			
6013.7274(2)	.72611	16628.6219	.6256	$\left({\;}^{2}P_{3/2}^{o}\right)5p$	^2^[3/2]_2_	–	$\left({\;}^{2}P_{3/2}^{o}\right)9s$	${\;}^{2}{\left[3/2\right]}_{2}^{o}$	3.7			
6013.8206(2)	.81950	16628.3644	.3674	$\left({\;}^{2}P_{3/2}^{o}\right)5p$	^2^[1/2]_1_	–	$\left({\;}^{2}P_{3/2}^{o}\right)6d$	${\;}^{2}{\left[3/2\right]}_{2}^{o}$	3.0			
6037.50529(10)	.50415	16563.1325	.1356	$\left({\;}^{2}P_{3/2}^{o}\right)5p$	^2^[3/2]_1_	–	$\left({\;}^{2}P_{3/2}^{o}\right)7d$	${\;}^{2}{\left[5/2\right]}_{2}^{o}$	3.1			
6057.80312(10)	.80211	16507.6345	.6373	$\left({\;}^{2}P_{3/2}^{o}\right)5p$	^2^[1/2]_1_	–	$\left({\;}^{2}P_{3/2}^{o}\right)6d$	${\;}^{2}{\left[1/2\right]}_{1}^{o}$	2.8			
6076.93707(10)	.93635	16455.6583	.6603	$\left({\;}^{2}P_{3/2}^{o}\right)5p$	^2^[3/2]_2_	–	$\left({\;}^{2}P_{3/2}^{o}\right)7d$	${\;}^{2}{\left[5/2\right]}_{3}^{o}$	2.0			
6084.54518(10)	.54409	16435.0822	.0851	$\left({\;}^{2}P_{3/2}^{o}\right)5p$	^2^[1/2]_1_	–	$\left({\;}^{2}P_{3/2}^{o}\right)6d$	${\;}^{2}{\left[1/2\right]}_{0}^{o}$	2.9			
6093.5139(2)	.51242	16410.8922	.8962	$\left({\;}^{2}P_{3/2}^{o}\right)5p$	^2^[3/2]_1_	–	$\left({\;}^{2}P_{3/2}^{o}\right)7d$	${\;}^{2}{\left[3/2\right]}_{2}^{o}$	4.0			
6110.0094(4)	.0082	16366.5869	.5902	$\left({\;}^{2}P_{3/2}^{o}\right)5p$	^2^[3/2]_1_	–	$\left({\;}^{2}P_{3/2}^{o}\right)7d$	${\;}^{2}{\left[1/2\right]}_{0}^{o}$	3.3			
6153.10907(10)	.10794	16251.9466	.9496	$\left({\;}^{2}P_{3/2}^{o}\right)5p$	^2^[3/2]_2_	–	$\left({\;}^{2}P_{3/2}^{o}\right)7d$	${\;}^{2}{\left[3/2\right]}_{2}^{o}$	3.0			
6165.3817(2)	.38052	16219.5959	.5991	$\left({\;}^{2}P_{3/2}^{o}\right)5p$	^2^[3/2]_1_	–	$\left({\;}^{2}P_{3/2}^{o}\right)7d$	${\;}^{2}{\left[1/2\right]}_{1}^{o}$	3.2			
6224.45490(10)	.45402	16065.6638	.6661	$\left({\;}^{2}P_{3/2}^{o}\right)5p$	^2^[5/2]_2_	–	$\left({\;}^{2}P_{3/2}^{o}\right)8s$	${\;}^{2}{\left[3/2\right]}_{1}^{o}$	2.3			
6238.07680(10)	.07589	16030.5817	.5841	$\left({\;}^{2}P_{3/2}^{o}\right)5p$	^2^[5/2]_3_	–	$\left({\;}^{2}P_{3/2}^{o}\right)8s$	${\;}^{2}{\left[3/2\right]}_{2}^{o}$	2.4			
6243.13125(10)	.13005	16017.6034	.6064	$\left({\;}^{2}P_{3/2}^{o}\right)5p$	^2^[5/2]_2_	–	$\left({\;}^{2}P_{3/2}^{o}\right)8s$	${\;}^{2}{\left[3/2\right]}_{2}^{o}$	3.0			
6348.43676(10)	.43559	15751.9093	.9122	$\left({\;}^{2}P_{3/2}^{o}\right)5p$	^2^[5/2]_3_	–	$\left({\;}^{2}P_{3/2}^{o}\right)6d$	${\;}^{2}{\left[5/2\right]}_{3}^{o}$	2.9			
6353.6716(2)	.67024	15738.9312	.9345	$\left({\;}^{2}P_{3/2}^{o}\right)5p$	^2^[5/2]_2_	–	$\left({\;}^{2}P_{3/2}^{o}\right)6d$	${\;}^{2}{\left[5/2\right]}_{3}^{o}$	3.3			
6370.0818(3)	.08059	15698.3855	.3885	$\left({\;}^{2}P_{3/2}^{o}\right)5p$	^2^[5/2]_3_	–	$\left({\;}^{2}P_{3/2}^{o}\right)6d$	${\;}^{2}{\left[5/2\right]}_{2}^{o}$	3.0			
6375.35220(10)	.35100	15685.4079	.4109	$\left({\;}^{2}P_{3/2}^{o}\right)5p$	^2^[5/2]_2_	–	$\left({\;}^{2}P_{3/2}^{o}\right)6d$	${\;}^{2}{\left[5/2\right]}_{2}^{o}$	3.0			
6411.9444(2)	.94347	15595.8932	.8955	$\left({\;}^{2}P_{3/2}^{o}\right)5p$	^2^[1/2]_0_	–	$\left({\;}^{2}P_{3/2}^{o}\right)7d$	${\;}^{2}{\left[3/2\right]}_{1}^{o}$	2.3			
6417.45257(10)	.45135	15582.5071	.5100	$\left({\;}^{2}P_{3/2}^{o}\right)5p$	^2^[5/2]_3_	–	$\left({\;}^{2}P_{3/2}^{o}\right)6d$	${\;}^{2}{\left[7/2\right]}_{3}^{o}$	2.9			
6422.80178(10)	.80048	15569.5292	.5324	$\left({\;}^{2}P_{3/2}^{o}\right)5p$	^2^[5/2]_2_	–	$\left({\;}^{2}P_{3/2}^{o}\right)6d$	${\;}^{2}{\left[7/2\right]}_{3}^{o}$	3.2			
6450.58162(10)	.58042	15502.4781	.4809	$\left({\;}^{2}P_{3/2}^{o}\right)5p$	^2^[5/2]_3_	–	$\left({\;}^{2}P_{3/2}^{o}\right)6d$	${\;}^{2}{\left[3/2\right]}_{2}^{o}$	2.8			
6458.07313(10)	.07189	15484.4948	.4978	$\left({\;}^{2}P_{3/2}^{o}\right)5p$	^2^[5/2]_3_	–	$\left({\;}^{2}P_{3/2}^{o}\right)6d$	${\;}^{2}{\left[7/2\right]}_{4}^{o}$	3.0(2)	2.80(7)^d^		
6489.86196(10)	.86103	15408.6482	.6504	$\left({\;}^{2}P_{3/2}^{o}\right)5p$	^2^[3/2]_1_	–	$\left({\;}^{2}P_{3/2}^{o}\right)8s$	${\;}^{2}{\left[3/2\right]}_{1}^{o}$	1.8			
6506.70156(10)	.70024	15368.7700	.7732	$\left({\;}^{2}P_{3/2}^{o}\right)5p$	^2^[5/2]_2_	–	$\left({\;}^{2}P_{3/2}^{o}\right)6d$	${\;}^{2}{\left[1/2\right]}_{1}^{o}$	3.2			
6510.1675(3)	.16628	15360.5879	.5908	$\left({\;}^{2}P_{3/2}^{o}\right)5p$	^2^[3/2]_1_	–	$\left({\;}^{2}P_{3/2}^{o}\right)8s$	${\;}^{2}{\left[3/2\right]}_{1}^{o}$	2.9			
6538.3575(2)	.35639	15294.3610	.3636	$\left({\;}^{2}P_{3/2}^{o}\right)5p$	^2^[3/2]_1_	–	$\left({\;}^{2}P_{3/2}^{o}\right)6d$	${\;}^{2}{\left[3/2\right]}_{2}^{o}$	2.6			
6557.3451(3)	.34388	15250.0743	.0771	$\left({\;}^{2}P_{3/2}^{o}\right)5p$	^2^[1/2]_0_	–	$\left({\;}^{2}P_{3/2}^{o}\right)7d$	${\;}^{2}{\left[1/2\right]}_{1}^{o}$	2.8			
6557.50560(10)	.50442	15249.7010	.7038	$\left({\;}^{2}P_{3/2}^{o}\right)5p$	^2^[3/2]_2_	–	$\left({\;}^{2}P_{3/2}^{o}\right)8s$	${\;}^{2}{\left[3/2\right]}_{1}^{o}$	2.8			
6578.23694(10)	.23583	15201.6416	.6441	$\left({\;}^{2}P_{3/2}^{o}\right)5p$	^2^[3/2]_2_	–	$\left({\;}^{2}P_{3/2}^{o}\right)8s$	${\;}^{2}{\left[3/2\right]}_{2}^{o}$	2.5			
6654.07167(10)	.07041	15028.3924	.3952	$\left({\;}^{2}P_{3/2}^{o}\right)5p$	^2^[3/2]_1_	–	$\left({\;}^{2}P_{3/2}^{o}\right)6d$	${\;}^{2}{\left[5/2\right]}_{2}^{o}$	2.8			
6701.07916(10)	.07793	14922.9695	.9723	$\left({\;}^{2}P_{3/2}^{o}\right)5p$	^2^[3/2]_2_	–	$\left({\;}^{2}P_{3/2}^{o}\right)6d$	${\;}^{2}{\left[5/2\right]}_{3}^{o}$	2.8			
6725.2001(3)	.19896	14869.4460	.4486	$\left({\;}^{2}P_{3/2}^{o}\right)5p$	^2^[3/2]_2_	–	$\left({\;}^{2}P_{3/2}^{o}\right)6d$	${\;}^{2}{\left[5/2\right]}_{2}^{o}$	2.6			
6741.9591(3)	.95742	14832.4839	.4876	$\left({\;}^{2}P_{3/2}^{o}\right)5p$	^2^[3/2]_1_	–	$\left({\;}^{2}P_{3/2}^{o}\right)6d$	${\;}^{2}{\left[3/2\right]}_{2}^{o}$	3.7			
6797.2858(3)	.28442	14711.7545	.7575	$\left({\;}^{2}P_{3/2}^{o}\right)5p$	^2^[3/2]_1_	–	$\left({\;}^{2}P_{3/2}^{o}\right)6d$	${\;}^{2}{\left[1/2\right]}_{1}^{o}$	3.0			
6814.98902(10)	.98762	14673.5380	.5410	$\left({\;}^{2}P_{3/2}^{o}\right)5p$	^2^[3/2]_2_	–	$\left({\;}^{2}P_{3/2}^{o}\right)6d$	${\;}^{2}{\left[3/2\right]}_{2}^{o}$	3.0			
6830.9733(3)	.97210	14639.2023	.2049	$\left({\;}^{2}P_{3/2}^{o}\right)5p$	^2^[3/2]_1_	–	$\left({\;}^{2}P_{3/2}^{o}\right)6d$	${\;}^{2}{\left[1/2\right]}_{0}^{o}$	2.6			
6848.2895(3)	.28879	14602.1864	.1879	$\left({\;}^{2}P_{3/2}^{o}\right)5p$	^2^[1/2]_1_	–	$\left({\;}^{2}P_{3/2}^{o}\right)7s$	${\;}^{2}{\left[3/2\right]}_{1}^{o}$	1.5			
6871.5262(3)	.52476	14552.8078	.8108	$\left({\;}^{2}P_{3/2}^{o}\right)5p$	^2^[3/2]_2_	–	$\left({\;}^{2}P_{3/2}^{o}\right)6d$	${\;}^{2}{\left[1/2\right]}_{1}^{o}$	3.0			
6906.5836(2)	.58254	14478.9386	.9408	$\left({\;}^{2}P_{3/2}^{o}\right)5s$	^2^[1/2]_1_	–	$\left({\;}^{2}P_{3/2}^{o}\right)7s$	${\;}^{2}{\left[3/2\right]}_{2}^{o}$	2.2			
7589.5025(2)	.50024	13176.0942	.0981	$\left({\;}^{2}P_{3/2}^{o}\right)5s$	${\;}^{2}{\left[3/2\right]}_{1}^{o}$	–	$\left({\;}^{2}P_{3/2}^{o}\right)5p$	^2^[1/2]_0_	3.9(4)	3.53(4)^k^		
7603.6384(2)	.63663	13151.5986	.6016	$\left({\;}^{2}P_{3/2}^{o}\right)5s$	${\;}^{2}{\left[3/2\right]}_{2}^{o}$	–	$\left({\;}^{2}P_{3/2}^{o}\right)5p$	^2^[3/2]_2_	3.0(4)	2.30(4)^k^		
7687.3612(2)	.3590	13008.3650	.3687	$\left({\;}^{2}P_{1/2}^{o}\right)5s$	${\;}^{2}{\left[1/2\right]}_{1}^{o}$	–	$\left({\;}^{2}P_{1/2}^{o}\right)5p$	^2^[1/2]_0_	3.7(3)	3.46(5)^k^		
7696.6579(2)	.65631	12992.6523	.6550	$\left({\;}^{2}P_{3/2}^{o}\right)5s$	${\;}^{2}{\left[3/2\right]}_{2}^{o}$	–	$\left({\;}^{2}P_{3/2}^{o}\right)5p$	^2^[3/2]_1_	2.7(2)	2.27(5)^k^		
7856.9844(2)	.98253	12727.5294	.5324	$\left({\;}^{2}P_{1/2}^{o}\right)5s$	${\;}^{2}{\left[1/2\right]}_{0}^{o}$	–	$\left({\;}^{2}P_{1/2}^{o}\right)5p$	^2^[1/2]_1_	3.0(3)	2.31(4)^k^		
7915.6020(2)	.60014	12633.2779	.2809	$\left({\;}^{2}P_{3/2}^{o}\right)5p$	^2^[1/2]_1_	–	$\left({\;}^{2}P_{3/2}^{o}\right)5d$	${\;}^{2}{\left[1/2\right]}_{1}^{o}$	3.0			
7930.7798(2)	.77813	12609.1006	.1032	$\left({\;}^{2}P_{3/2}^{o}\right)5p$	^2^[5/2]_2_	–	$\left({\;}^{2}P_{3/2}^{o}\right)5d$	${\;}^{2}{\left[7/2\right]}_{3}^{o}$	2.6			
8061.7211(5)	.71987	12404.2991	.3010	$\left({\;}^{2}P_{1/2}^{o}\right)5s$	${\;}^{2}{\left[1/2\right]}_{0}^{o}$	–	$\left({\;}^{2}P_{1/2}^{o}\right)5p$	^2^[3/2]_1_	1.9(8)	2.20(5)^k^		
8115.1320(2)	.12988	12322.6585	.6617	$\left({\;}^{2}P_{3/2}^{o}\right)5s$	${\;}^{2}{\left[3/2\right]}_{2}^{o}$	–	$\left({\;}^{2}P_{3/2}^{o}\right)5p$	^2^[5/2]_3_	3.2(3)	2.19(6)^k^		
8192.3082(3)	.30538	12206.5720	.5762	$\left({\;}^{2}P_{3/2}^{o}\right)5s$	${\;}^{2}{\left[3/2\right]}_{1}^{o}$	–	$\left({\;}^{2}P_{3/2}^{o}\right)5p$	${\;}^{2}{\left[3/2\right]}_{2}$	4.2(4)	3.35(4)^k^		
8265.5140(3)	.51129	12098.4611	.4651	$\left({\;}^{2}P_{1/2}^{o}\right)5s$	${\;}^{2}{\left[1/2\right]}_{1}^{o}$	–	$\left({\;}^{2}P_{1/2}^{o}\right)5p$	${\;}^{2}{\left[3/2\right]}_{2}$	4.0(4)	3.31(5)^k^		
8283.3284(2)	.32590	12072.4418	.4454	$\left({\;}^{2}P_{1/2}^{o}\right)5s$	${\;}^{2}{\left[1/2\right]}_{1}^{o}$	–	$\left({\;}^{2}P_{1/2}^{o}\right)5p$	${\;}^{2}{\left[1/2\right]}_{1}$	3.6(3)	3.24(5)^k^		
8300.3907(3)	.38801	12047.6257	.6296	$\left({\;}^{2}P_{3/2}^{o}\right)5s$	${\;}^{2}{\left[3/2\right]}_{1}^{o}$	–	$\left({\;}^{2}P_{3/2}^{o}\right)5p$	${\;}^{2}{\left[3/2\right]}_{1}$	3.9(4)	3.44(5)^k^		
8511.2106(4)	.20763	11749.2099	.2140	$\left({\;}^{2}P_{1/2}^{o}\right)5s$	${\;}^{2}{\left[1/2\right]}_{1}^{o}$	–	$\left({\;}^{2}P_{1/2}^{o}\right)5p$	${\;}^{2}{\left[3/2\right]}_{1}$	4.1(6)	3.27(5)^k^		
8779.1607(3)	.15807	11390.6105	.6139	$\left({\;}^{2}P_{3/2}^{o}\right)5s$	${\;}^{2}{\left[3/2\right]}_{1}^{o}$	–	$\left({\;}^{2}P_{3/2}^{o}\right)5p$	${\;}^{2}{\left[5/2\right]}_{2}$	3.4(4)	3.30(7)^k^		
8931.1447(2)	.14294	11196.7730	.7752	$\left({\;}^{2}P_{3/2}^{o}\right)5s$	${\;}^{2}{\left[3/2\right]}_{2}^{o}$	–	$\left({\;}^{2}P_{3/2}^{o}\right)5p$	${\;}^{2}{\left[1/2\right]}_{1}$	2.2			
9754.4352(4)	.43235	10251.7468	.7498	$\left({\;}^{2}P_{3/2}^{o}\right)5s$	${\;}^{2}{\left[3/2\right]}_{1}^{o}$	–	$\left({\;}^{2}P_{3/2}^{o}\right)5p$	${\;}^{2}{\left[1/2\right]}_{1}$	3.0			

a This is the uncertainty in the Kr^84^ wavelength.

b Wavenumber of the Kr^86^ transition minus that of the Kr^84^ transition in units of 10^−3^ cm^−1^. The uncertainty of the last digit of the Kr^84^ measurement is given in parentheses for those transitions for which direct isotope shift measurements are available.

c In units of 10^−3^ cm^−1^.

d Direct (Kr^86^–Kr^84^) isotope shift measurement by Ref. [6]. The uncertainty of the measurement is given in parentheses.

e Direct (Kr^86^–Kr^84^) isotope shift measurement by Ref. [7]. The uncertainty of the measurement is given in parentheses.

f Direct (Kr^86^–Kr^84^) isotope shift measurement by Ref. [8]. The uncertainty of the measurement is given in parentheses.

g Direct (Kr^86^–Kr^84^) isotope shift measurement by Ref. [9]. The uncertainty of the measurement is given in parentheses.

h Direct (Kr^86^–Kr^84^) isotope shift measurement by Ref. [10]. The uncertainty of the measurement is given in parentheses.

i Direct (Kr^86^–Kr^84^) isotope shift measurement by Ref. [11]. The uncertainty of the measurement is given in parentheses.

j Direct (Kr^86^–Kr^84^) isotope shift measurement by Ref. [12]. The uncertainty of the measurement is given in parentheses.

k Direct (Kr^86^–Kr^84^) isotope shift measurement by Ref. [13]. The uncertainly of the measurement is given in parentheses.

**Table 2:** Energy level of Kr^84^ I in units of cm^−1^

Energy level value		Δ*ν*_lev_^a^	Δ*ν*_nm_	Configuration	Level_J_	*N*^b^
Kr^84^	Kr^86^					
0.0000(62)	.0000(62)	0.0	17.0	4*s*^2^4*p*^6^	^1^S_0_	3
79971.7301(3)	.7321(1)	2.0	5.0	$4s^{2}4p^{5}\left({\;}^{2}P_{3/2}^{o}\right)5s$	${\;}^{2}{\left[3/2\right]}_{2}^{o}$	12
80916.7564(3)	.7575(1)	1.1	4.8		${\;}^{2}{\left[3/2\right]}_{1}^{o}$	12
85191.6050(5)	.6075(1)	2.5	5.0	$4s^{2}4p^{5}\left({\;}^{2}P_{1/2}^{o}\right)5s$	${\;}^{2}{\left[1/2\right]}_{0}^{o}$	6
85846.6930(4)	.6945(1)	1.5	4.9		${\;}^{2}{\left[1/2\right]}_{1}^{o}$	14
91168J034(3)	.5073(1)	3.9	3.2	$4s^{2}4p^{5}\left({\;}^{2}P_{3/2}^{o}\right)5p$	^2^[1/2]_1_	15
94092.8510(6)	.8557(1)	4.7	2.8		^2^[1/2]_0_	3
92294.3896(3)	.3938(1)	4.2	3.1	$4s^{2}4p^{5}\left({\;}^{2}P_{3/2}^{o}\right)5p$	^2^[5/2]_3_	14
92307.3670(3)	.3714(1)	4.4	3.1		^2^[5/2]_2_	14
929643827(3)	.3871(1)	4.4	3.0	$4s^{2}4p^{5}\left({\;}^{2}P_{3/2}^{o}\right)5p$	^2^[3/2]_1_	15
93123.3293(3)	.3337(1)	4.4	3.0		^2^[3/2]_2_	14
97595.9037(6)	.9086(2)	4.9	3.1	$4s^{2}4p^{5}\left({\;}^{2}P_{1/2}^{o}\right)5p$	^2^[3/2]_1_	4
97945.1548(6)	.1597(2)	4.9	3.0		^2^[3/2]_2_	3
97919.1352(5)	.1400(1)	4.8	3.0	$4s^{2}4p^{5}\left({\;}^{2}P_{1/2}^{o}\right)5p$	^2^[1/2]_1_	4
98855.0582(10)	.0632(3)	5.0	2.9		^2^[1/2]_0_	1
102887.1820(6)	.1878(1)	5.8	1.5	$4s^{2}4p^{5}\left({\;}^{2}P_{3/2}^{o}\right)6p$	^2^[1/2]_1_	3
103761.6220(7)	.6280(2)	6.0	1.4		^2^[1/2]_0_	2
103115.6227(16)	.6286(4)	5.9	1.4	$4s^{2}4p^{5}\left({\;}^{2}P_{3/2}^{o}\right)6p$	^2^[5/2]_3_	1
103121.1303(12)	.1362(2)	5.9	1.4		^2^[5/2]_2_	2
103313.4610(6)	.4669(2)	5.9	1.4	$4s^{2}4p^{5}\left({\;}^{2}P_{3/2}^{o}\right)6p$	^2^[3/2]_1_	3
103362.6008(6)	.6067(3)	5.9	1.4		^2^[3/2]_2_	3
103801.7813(10)	.7882(1)	6.9	1.3	$4s^{2}4p^{5}\left({\;}^{2}P_{3/2}^{o}\right)5d$	${\;}^{2}{\left[1/2\right]}_{1}^{o}$	1
104916.4676(10)	.4746(2)	7.0	1.2	$4s^{2}4p^{5}\left({\;}^{2}P_{3/2}^{o}\right)5d$	${\;}^{2}{\left[7/2\right]}_{3}^{o}$	1
105647.4420(10)	.4482(2)	6.2	1.0	$4s^{2}4p^{5}\left({\;}^{2}P_{3/2}^{o}\right)7s$	${\;}^{2}{\left[3/2\right]}_{2}^{o}$	1
105770.6898(10)	.6953(1)	5.5	1.0		${\;}^{2}{\left[3/2\right]}_{1}^{o}$	2
107603.5853(7)	.5924(2)	7.1	0.8	$4s^{2}4p^{5}\left({\;}^{2}P_{3/2}^{o}\right)6d$	${\;}^{2}{\left[1/2\right]}_{0}^{o}$	2
107676.1373(5)	.1446(1)	7.3	0.8		${\;}^{2}{\left[1/2\right]}_{1}^{o}$	4
107778.8846(10)	.8916(1)	7.0	0.7	$4s^{2}4p^{5}\left({\;}^{2}P_{3/2}^{o}\right)6d$	${\;}^{2}{\left[7/2\right]}_{4}^{o}$	1
107876.8964(7)	.9038(2)	7.4	0.7		${\;}^{2}{\left[7/2\right]}_{3}^{o}$	2
107796.8674(5)	.8747(2)	7.3	0.7	$4s^{2}4p^{5}\left({\;}^{2}P_{3/2}^{o}\right)6d$	${\;}^{2}{\left[3/2\right]}_{2}^{o}$	4
108258.7437(10)	.7507(2)	7.0	0.7		${\;}^{2}{\left[3/2\right]}_{1}^{o}$	1
107992.7751(5)	.7823(1)	7.2	0.7	$4s^{2}4p^{5}\left({\;}^{2}P_{3/2}^{o}\right)6d$	${\;}^{2}{\left[5/2\right]}_{2}^{o}$	4
108046.2986(6)	.3060(1)	7.4	0.7		${\;}^{2}{\left[5/2\right]}_{3}^{o}$	3
108324.9706(5)	.9779(1)	7.3	0.7	$4s^{2}4p^{5}\left({\;}^{2}P_{3/2}^{o}\right)8s$	${\;}^{2}{\left[3/2\right]}_{2}^{o}$	4
108373.0307(6)	.0375(1)	6.8	0.7		${\;}^{2}{\left[3/2\right]}_{1}^{o}$	3
108438.2487(7)	.2555(2)	6.8	1.4	$4s^{2}4p^{5}\left({\;}^{2}P_{1/2}^{o}\right)6p$	^2^[3/2]_1_	2
108567.7573(10)	.7650(4)	7.7	1.4		^2^[3/2]_2_	1
108514.1708(7)	.1776(2)	6.8	1.4	$4s^{2}4p^{5}\left({\;}^{2}P_{1/2}^{o}\right)6p$	^2^[1/2]_1_	2
108821.5574(10)	.5639(3)	6.5	1.4		^2^[1/2]_0_	1
108471.1161(16)	.1225(5)	6.4	0.6	$4s^{2}4p^{5}\left({\;}^{2}P_{3/2}^{o}\right)5f$	^2^[3/2]_2_	1
109296.1801(17)	.1863(5)	6.2	0.5	$4s^{2}4p^{5}\left({\;}^{2}P_{3/2}^{o}\right)8p$	^2^[1/2]_0_	1
109330.9706(9)	.9773(7)	6.7	0.5	$4s^{2}4p^{5}\left({\;}^{2}P_{3/2}^{o}\right)7d$	${\;}^{2}{\left[1/2\right]}_{0}^{o}$	2
109342.9252(6)	.9328(2)	7.6	0.5		${\;}^{2}{\left[1/2\right]}_{1}^{o}$	3
109375.2759(6)	.2833(2)	7.4	0.5	$4s^{2}4p^{5}\left({\;}^{2}P_{3/2}^{o}\right)7d$	${\;}^{2}{\left[3/2\right]}_{2}^{o}$	4
109688.7442(7)	.7511(2)	6.9	0.5		${\;}^{2}{\left[3/2\right]}_{1}^{o}$	2
109433.8963(10)	.9038(2)	7.5	0.5	$4s^{2}4p^{5}\left({\;}^{2}P_{3/2}^{o}\right)7d$	${\;}^{2}{\left[7/2\right]}_{4}^{o}$	1
109471.4098(7)	.4165(3)	6.7	0.5		${\;}^{2}{\left[7/2\right]}_{3}^{o}$	2
109527.5152(7)	.5227(2)	7.5	0.5	$4s^{2}4p^{5}\left({\;}^{2}P_{3/2}^{o}\right)7d$	${\;}^{2}{\left[5/2\right]}_{2}^{o}$	2
109578.9874(8)	.9940(5)	6.6	0.5		${\;}^{2}{\left[5/2\right]}_{3}^{o}$	2
109751.9523(7)	.9593(3)	7.0	0.4	$4s^{2}4p^{5}\left({\;}^{2}P_{3/2}^{o}\right)9s$	${\;}^{2}{\left[3/2\right]}_{2}^{o}$	3
109779.3024(8)	.3081(3)	5.7	0.4		${\;}^{2}{\left[3/2\right]}_{1}^{o}$	2
110103.7771(8)	.2299(5)	7.6	1.2	$4s^{2}4p^{5}\left({\;}^{2}P_{1/2}^{o}\right)5d$	${\;}^{2}{\left[3/2\right]}_{2}^{o}$	2
110121.9845(10)	.9917(9)	7.2	1.2	$4s^{2}4p^{5}\left({\;}^{2}P_{1/2}^{o}\right)5d$	${\;}^{2}{\left[5/2\right]}_{2}^{o}$	1
110290.3049(10)	.3120(2)	7.1	0.4	$4s^{2}4p^{5}\left({\;}^{2}P_{3/2}^{o}\right)8d$	${\;}^{2}{\left[1/2\right]}_{1}^{o}$	1
110335.6153(12)	.6214(7)	6.1	0.4		${\;}^{2}{\left[1/2\right]}_{0}^{o}$	1
110403.6269(10)	.6333(2)	6.4	0.3	$4s^{2}4p^{5}\left({\;}^{2}P_{3/2}^{o}\right)8d$	${\;}^{2}{\left[7/2\right]}_{4}^{o}$	1
110470.9073(10)	.9140(7)	6.7	0.3		${\;}^{2}{\left[7/2\right]}_{3}^{o}$	2
110496.7006(10)	.7083(12)	7.7	0.3	$4s^{2}4p^{5}\left({\;}^{2}P_{3/2}^{o}\right)8d$	${\;}^{2}{\left[5/2\right]}_{2}^{o}$	1
111018.8656(10)	.8713(6)	5.7	0.3	$4s^{2}4p^{5}\left({\;}^{2}P_{3/2}^{o}\right)9d$	${\;}^{2}{\left[7/2\right]}_{4}^{o}$	1
111047.1577(11)	.1626(13)	4.9	0.2		${\;}^{2}{\left[7/2\right]}_{3}^{o}$	1

a The difference between the level values of the two isotopes is given in units of 10^−3^ cm^−1^.

b The number in this column indicates the number of intcrferometrically measured lines with transitions to or from that level.

**Table 3:** Energy level values of Kr^82^, Kr^80^, and Kr^78^ in units of cm^−1^ and their differences and normal mass shifts with respect *to those* of Kr^86^ in units of 10^−3^ cm^−1^

Config.	Level	Energy level value			Kr^86^–Kr^82^		Kr^86^–Kr^80^		Kr^86^–Kr^78^	
		Kr^82^	Kr^80^	Kr^78^	Δ*ν*_lev_	Δ*ν*_nm_	Δ*ν*_lev_	Δ*ν*_nm_	Δ*ν*_lev_	Δ*ν*_nm_
4*p*^6^	IS_0_	0.0000	.0000	.0000	0.0	34.9	0.0	53.6	0.0	73.3
$\left({\;}^{2}P_{3/2}^{o}\right)5s$	${\;}^{2}{\left[3/2\right]}_{2}^{o}$	79971.7275	.7252	.7232	4.6	10.2	6.9	15.6	8.9	21.4
$\left({\;}^{2}P_{3/2}^{o}\right)5s$	${\;}^{2}{\left[3/2\right]}_{1}^{o}$	80916.7549	.7535	.7528	2.6	9.9	4.0	15.2	4.7	20.8
$\left({\;}^{2}P_{1/2}^{o}\right)5s$	${\;}^{2}{\left[1/2\right]}_{0}^{o}$	85191.6019	.5991	.5964	5.6	10.2	8.4	15.7	11.1	21.5
$\left({\;}^{2}P_{1/2}^{o}\right)5s$	${\;}^{2}{\left[1/2\right]}_{1}^{o}$	85846.6910	.6892	.6876	3.5	10.0	5.3	15.4	6.9	21.1
$\left({\;}^{2}P_{3/2}^{o}\right)5p$	^2^[1/2]_0_	94092.8460	.8408	.8361	9.7	5.8	14.9	8.9	19.6	12.2
$\left({\;}^{2}P_{3/2}^{o}\right)5p$	^2^[5/2]_3_	92294.3849	.3801	.3756	8.9	6.4	13.7	9.8	18.2	13.4
$\left({\;}^{2}P_{3/2}^{o}\right)5p$	^2^[5/2]_2_	92307.3623	.3573	.3527	9.1	6.4	14.1	9.8	18.7	13.4
$\left({\;}^{2}P_{3/2}^{o}\right)5p$	^2^[3/2]_1_	92964.3778	.3728	.3680	*93*	6.2	14.3	9.5	19.1	13.0
$\left({\;}^{2}P_{3/2}^{o}\right)5p$	^2^[3/2]_2_	93123.3244	.3195	.3147	9.3	6.1	14.2	9.4	19.0	12.9
$\left({\;}^{2}P_{1/2}^{o}\right)5p$	^2^[3/2]_1_	97595.8985	.8931	.8877	10.1	6.4	15.5	9.8	20.9	13.4
$\left({\;}^{2}P_{1/2}^{o}\right)5p$	^2^[3/2]_2_	97945.1495	.1440	.1388	10.2	6.3	15.7	9.7	20.9	13.2
$\left({\;}^{2}P_{1/2}^{o}\right)5p$	^2^[1/2]_1_	97919.1298	.1244	.1189	10.2	6.3	15.6	9.7	21.1	13.2
$\left({\;}^{2}P_{1/2}^{o}\right)5p$	^2^[1/2]_0_	98855.0528	.0471	.0416	10.4	6.0	16.1	9.2	21.6	12.6
$\left({\;}^{2}P_{3/2}^{o}\right)6p$	^2^[1/2]_1_	102887.1757	.1693	.1629	12.1	3.1	18.5	4.8	24.9	6.5
$\left({\;}^{2}P_{3/2}^{o}\right)6p$	^2^[1/2]_0_	103761.6158	.6088	.6025	12,2	2.8	19.2	4.3	25.5	5.9
$\left({\;}^{2}P_{3/2}^{o}\right)6p$	^2^[5/2]_3_	103115.6164	.6099	.6033	12.2	3.0	18.7	4.7	25.3	6.4
$\left({\;}^{2}P_{3/2}^{o}\right)6p$	^2^[5/2]_2_	103121.1243	.1174	.1112	11.9	3.0	18.8	4.6	25.0	6.4
$\left({\;}^{2}P_{3/2}^{o}\right)6p$	^2^[3/2]_1_	103313.4548	.4479	.4416	12.1	3.0	19.0	4.6	25.3	6.2
$\left({\;}^{2}P_{3/2}^{o}\right)6p$	^2^[3/2]_2_	103362.5944	.5877	.5811	12.3	3.0	19.0	4.5	25.6	6.2
$\left({\;}^{2}P_{3/2}^{o}\right)6d$	${\;}^{2}{\left[7/2\right]}_{4}^{o}$	107778.8771	.8693	.8616	14.5	1.6	22.3	2.4	30.0	3.3

**Table 4:** Term-value shifts, in units of 10^−3^ cm^−1^, due to the sum of the specific mass and volume effects

Config.	Term	Kr^86^–Kr^84^	Kr^86^–Kr^82^	Kr^86^–Kr^80^	Kr^86^–Kr^78^
4*p*^6^	^1^S_0_	−9.5	−19.3	−29.5	−40.9
$\left({\;}^{2}P_{3/2}^{o}\right)5s$	${\;}^{2}{\left[3/2\right]}_{2}^{o}$	0.5	0.8	1.6	1.9
$\left({\;}^{2}P_{3/2}^{o}\right)5s$	${\;}^{2}{\left[3/2\right]}_{1}^{o}$	1.6	3.1	4.9	6.9
$\left({\;}^{2}P_{1/2}^{o}\right)5s$	${\;}^{2}{\left[1/2\right]}_{0}^{o}$	0.0	−0.2	0.0	−0.2
$\left({\;}^{2}P_{1/2}^{o}\right)5s$	${\;}^{2}{\left[1/2\right]}_{1}^{o}$	1.1	2.1	3.4	4.4
$\left({\;}^{2}P_{3/2}^{o}\right)5p$	^2^[1/2]_0_	0.0	0.1	0.3	0.6
$\left({\;}^{2}P_{3/2}^{o}\right)5p$	^2^[5/2]_3_	0.2	0.3	0.6	0.8
$\left({\;}^{2}P_{3/2}^{o}\right)5p$	^2^[5/2]_2_	0.0	0.1	0.2	0.3
$\left({\;}^{2}P_{3/2}^{o}\right)5p$	^2^[3/2]_1_	0.1	0.1	0.3	0.3
$\left({\;}^{2}P_{3/2}^{o}\right)5p$	^2^[3/2]_2_	0.1	0.2	0.5	0.5
$\left({\;}^{2}P_{1/2}^{o}\right)5p$	^2^[3/2]_1_	−0.5	−0.9	−1.2	−1.9
$\left({\;}^{2}P_{1/2}^{o}\right)5p$	^2^[3/2]_2_	−0.6	−0.9	−1.3	−1.7
$\left({\;}^{2}P_{1/2}^{o}\right)5p$	^2^[1/2]_1_	−0.3	−0.9	−1.2	−1.9
$\left({\;}^{2}P_{1/2}^{o}\right)5p$	^2^[1/2]_0_	−0.4	−0.8	−1.2	−1.8
$\left({\;}^{2}P_{3/2}^{o}\right)6p$	^2^[1/2]_1_	0.2	0.4	0.8	1.0
$\left({\;}^{2}P_{3/2}^{o}\right)6p$	^2^[1/2]_0_	0.1	0.6	0.6	1.0
$\left({\;}^{2}P_{3/2}^{o}\right)6p$	^2^[5/2]_3_	0.2	0.4	0.7	0.7
$\left({\;}^{2}P_{3/2}^{o}\right)6p$	^2^[5/2]_2_	0.2	0.7	0.7	1.0
$\left({\;}^{2}P_{3/2}^{o}\right)6p$	^2^[3/2]_1_	0.2	0.5	0.5	0.9
$\left({\;}^{2}P_{3/2}^{o}\right)6p$	^2^[3/2]_2_	0.2	0.3	0.6	0.6
$\left({\;}^{2}P_{3/2}^{o}\right)6d$	${\;}^{2}{\left[7/2\right]}_{4}^{o}$	−0.2	−0.6	−0.6	−0.9
